# Norms for gender stereotypically congruent, stereotypically incongruent, semantically correct, and semantically incorrect sentences in Polish and English

**DOI:** 10.1371/journal.pone.0302594

**Published:** 2024-05-16

**Authors:** Katarzyna Jankowiak, Marcin Naranowicz, Anna Skałba, Dariusz Drążkowski, Joanna Pawelczyk

**Affiliations:** 1 Faculty of English, Adam Mickiewicz University, Poznań, Poland; 2 Faculty of Psychology and Cognitive Sciences, Adam Mickiewicz University, Poznań, Poland; Universidad Complutense Madrid, SPAIN

## Abstract

The present contribution provides ratings for a database of gender stereotypically congruent, stereotypically incongruent, semantically correct, and semantically incorrect sentences in Polish and English. A total of 942 volunteers rated 480 sentences (120 per condition) in each language in terms of their meaningfulness, probability of use, and stereotypicality. The stimuli were highly controlled for their length and critical words, which were shared across the conditions. The results of the ratings revealed that stereotypically incongruent sentences were consciously evaluated as both less meaningful and probable to use relative to sentences that adhere to stereotype-driven expectations regarding males and females, indicating that stereotype violations communicated through language exert influence on language perception. Furthermore, the results yielded a stronger internalization of gender stereotypes among sex-typed individuals, thus pointing to the crucial role of gender schema in the sensitivity to gender stereotypical attributes. The ratings reported in the present article aim to broaden researchers’ stimulus choices and allow for consistency across different laboratories and research projects on gender stereotype processing. The adaptation of this database to other languages or cultures could also enable a cross-cultural comparison of empirical findings on stereotype processing.

## 1. Introduction

Gender stereotypes are defined as broad generalizations about social norms and expectations assigned to males and females [1], and are assumed to derive from the discrepant distribution of males and females engaged in the social roles they perform at home and work (*social role theory* [2,3]). While gender stereotypes can serve an adaptive function, facilitating categorization and anticipation processes regarding others [4,5], they often induce an incorrect assessment of other people’s skills and unique qualities, thus positively or negatively affecting expectations we generate towards someone’s performance [6,7]. Importantly, gender stereotypes are strongly transmitted and reinforced through language [8,9]. Previous research has consistently shown that gender stereotypes are automatically accessed in the process of language comprehension and are difficult to suppress [10–12]. While much attention has been devoted to the role of single role nouns (e.g., *nurse*, *scientist*) in perpetuating gender stereotypes [13], the present contribution aims to provide the first normed dataset of sentences that either align with or violate gender stereotypes, showing how the perception of such sentences can uncover stereotypical thinking, even in the absence of role nouns.

Previous neurophysiological research (e.g., [14,15]) has pointed to more cognitively taxing mechanisms engaged when processing sentences that violate gender stereotypes (e.g., *The notary is breastfeeding*. [14]) relative to those that accord with gender-related expectations (e.g., *The chemist put on a nice tie*. [14]). The processing of both types of sentences requires the activation of stereotype knowledge stored in long-term memory, which is then integrated with the semantic meaning of the sentence [14]. While stereotypically congruent sentences reflect the patterns that are stored in long-term memory based on their frequency of occurrence, stereotypically incongruent sentences require a more robust activation of memory networks, as they violate the frequency-driven expectations regarding males and females. This, in turn, increases the cognitive load that stereotypically incongruent meanings evoke, as reflected in longer reaction times as well as increased physiological responding to such sentences [10,12,13,16,17]. Interestingly, the effect has also been replicated in survey research [18,19].

In order to investigate how the brain processes gender stereotypes expressed in language, psycholinguistic research employs linguistic materials that represent stimuli that are stereotypically congruent (those that adhere to the expectations or norms typically associated with a particular gender) and stereotypically incongruent (those that violate the expectations or norms typically associated with a particular gender) [10,14]. Taking into account the number of factors that influence word processing (e.g., word frequency, concreteness, length, or emotional value), it seems crucial that the two categories of sentences are as close to one another as possible, so that the only factor being manipulated is the stereotype (in)congruency. The present database therefore provides a set of stereotypically congruent (e.g., *Females* *carefully watch their* ***waistline*** *before the wedding*.) and stereotypically incongruent sentences (e.g., *Males* *carefully watch their* ***waistline*** *before the wedding*.). In such sentences, the critical word (i.e., *waistline*) is shared across the two conditions, thus enabling the control over the aforementioned linguistic factors. It is consequently the very beginning of the sentence that creates stereotype-driven expectations, while the critical word is used to either affirm or disprove them.

Furthermore, given that cognitive mechanisms engaged in sentence processing are highly modulated by language meaningfulness and probability of use (see [20] for a review), each sentence included in the present database was also controlled and evaluated for these two factors. Namely, we expect that while both stereotypically congruent and stereotypically incongruent sentences represent meaningful conditions, their probability of use should differ considerably, with stereotypically incongruent meanings being evaluated as less probable to be encountered in everyday language. Such a low probability of use of such sentences additionally increases their processing difficulty, which might be reflected in lower meaningfulness ratings.

Importantly, the present database adds a unique contribution to the research on gender stereotype processing. Prior studies (e.g., [10,13,14]), have typically only compared stereotypically congruent versus incongruent sentences. In contrast, our contribution goes beyond this binary approach by also including two additional conditions: semantically correct and semantically incorrect sentences. The decision to include those two sentence types in the present database was driven by two factors. First, adding these two conditions enables the comparison of stereotypically congruent and incongruent sentences across the meaningfulness continuum. This allows the investigation of whether and to what extent stereotypically incongruent sentences are evaluated as less semantically correct relative to stereotypically congruent sentences, which should be evaluated as high in meaningfulness. Second, the inclusion of semantically correct and incorrect sentences is particularly relevant for behavioral and psychophysiological methodology, where differences between semantically correct and incorrect items are typically used as points of reference when investigating lexico-semantic effects on reaction times and/or event-related potential (ERP) components [10,12,13]. Thus, by using all four sentence types, our database provides a comprehensive approach to examining gender stereotype processing, while also allowing for the replication of the well-established findings from behavioral and psychophysiological research.

Finally, special research attention has recently been devoted to dissociating the effects of biological sex and gender role (i.e., societal expectations about the behaviors, attitudes, and traits considered appropriate for one’s sex and gender), which may also play a pivotal role in gender stereotype perception [1]. According to the gender schema theory [23], individuals are inclined to organize, perceive, process, select, and memorize sex-typed information based on their own gender schema, i.e. cognitive structures that organize and process information related to gender. Particularly, sex-typed individuals, who strongly adhere to their expected male and female gender roles (females with dominant women’s gender schemas and males with dominant men’s gender schemas), are assumed to be more predisposed to use gender schemas to organize and process sex-typed information about themselves and others. In contrast, those that either violate their expected gender roles, i.e., individuals that display traits of their opposite gender (i.e., cross-sex-typed individuals), those with high levels of both masculine and feminine traits (i.e., androgynous individuals), or those displaying low levels of both of those features (i.e., undifferentiated individuals), are more likely to showcase a decreased sensitivity to stereotypically masculine and feminine features [23]. Consequently, the perception of gender stereotypes encoded in language is assumed to be modulated by individuals’ gender schema, with the highest sensitivity to stereotypical features observed in sex-typed individuals.

For instance, recent electrophysiological evidence has suggested that gender roles may determine one’s emotional responding instead of their biological sex, with feminine relative to masculine individuals being more susceptible to emotional content regardless of their sex [21]. Others have also observed that an individual’s perception of their own empathy may be mediated by gender role, with feminine gender-role being associated with a greater degree of self-reported empathy [22]. To extend these findings, in the present contribution, we provide further insights into the relationship between gender, gender role, and gender stereotype perception (e.g., [1,23,24]), as manifested in the perception of linguistic stereotype-(in)congruent stimuli.

In the following sections, we provide the ratings for a database of Polish (Study I) and English (Study II) gender stereotypically congruent and incongruent sentences as well as semantically correct and incorrect sentences (baseline conditions), as assessed in terms of their meaningfulness, probability of use, and stereotypicality. We also show whether and how respondents’ individual differences (i.e., gender and gender schema) modulate raters’ perception of the stimuli. The ratings reported in the present article aim to broaden researchers’ stimulus choices and allow for consistency across different laboratories and research projects on gender stereotype processing.

## 2. Study I: The perception of Polish sentences

### 2.1. Methods

#### 2.1.1. Participants

The original sample included 507 native speakers of Polish (257 females, 250 males), 35 (7.42%) of whom were excluded due to providing incomplete answers. Accordingly, statistical analyses were based on responses from 472 participants (240 females, 232 males), distributed across three surveys (see section 2.1.3 for more details): the meaningfulness (177 participants; 90 females, 87 males), probability of use (148 participants; 75 females, 73 males), and stereotypicality surveys (147 participants; 75 females, 72 males). Participants’ ages ranged from 18 to 40 years old (*M* = 25.36 years, *SD* = 4.18; meaningfulness: *M* = 25.48, *SD* = 4.20; probability of use: *M* = 25.35, *SD* = 4.30; stereotypicality: *M* = 25.22, *SD* = 4.07). Overall, almost half of the participants (45.76%) completed high school at most, over one fourth (28.39%) had a bachelor’s degree, and one fifth (20.97%) had a master’s degree.

#### 2.1.2. Stimuli

The stimuli designed for Study 1 consisted of 480 declarative sentences, divided into four categories: 120 stereotypically congruent (e.g., *Their niece became a* *hairdresser* *immediately after graduating*.), 120 stereotypically incongruent (e.g., *Their nephew became a* *hairdresser* *immediately after graduating*.), 120 semantically correct (e.g., *There is only one* *hairdresser* *with such experience*.), and 120 semantically incorrect sentences (e.g., *The cooks seasoned the* *hairdresser* *with fresh chili*.). The two gender-stereotyped conditions (stereotypically congruent and incongruent sentences) featured 50% of female stereotype-biased and 50% of male stereotype-biased sentences. The sentences were controlled for length (*M* = 8.0; range: 7–9), and included a stereotypically feminine (*n* = 60 per sentence type) or masculine (*n* = 60 per sentence type) critical noun placed in a mid-sentence position (i.e., always as the third word from the end). The critical nouns were selected so as to stereotypically, yet not explicitly, activate a male or female referent; a doll; a car). The critical words were all matched on length, the number of letters and syllables, frequency (SUBTLEX-PL [25]), valence, arousal, concreteness, and age of acquisition [26].

#### 2.1.3. Procedure

The present study was conducted as a part of a research project approved by the Ethics Committee for Research Involving Human Participants at Adam Mickiewicz University, Poznań (Resolution No. 12/2021/2022). All participants provided their written informed consent before taking part in the study. Ratings were collected using online surveys (SurveyMonkey, Momentive Inc.) between May and September 2022. Each participant was randomly assigned to one type of the surveys: the meaningfulness, probability of use, or stereotypicality surveys. Each survey took approximately 10 minutes to complete.

Unlike the meaningfulness survey, which included all the four sentence types (i.e., stereotypically congruent, stereotypically incongruent, semantically correct, and semantically incorrect sentences), the probability of use and stereotypicality surveys comprised three sentence types (i.e., stereotypically congruent, stereotypically incongruent, and semantically correct sentences). The stimuli were divided into four (the meaningfulness survey) or three lists (the probability of use and stereotypical congruency surveys), each consisting of 120 randomly presented sentences, with an equal proportion of sentences per condition. Each survey was completed by a minimum of 27–30 participants (13–15 males and 13–15 females).

In the meaningfulness survey, participants were asked to decide how meaningful or meaningless a given sentence was on a 7-point Likert scale (1 –*very meaningless*, 7 –*very meaningful*). Please note that in the present study, we operationally defined the *meaningfulness* dimension as reflecting concepts that were either in line with semantic knowledge or violated it. Participants’ ratings were thus based on the extent to which a given sentence adhered to what they assumed to be semantically correct, aligning with their conceptual knowledge about the world. Similarly, sentences categorized as meaningless (i.e., the semantically incorrect condition) were considered anomalous as they contradicted participants’ conceptual knowledge, thus reflecting semantic violations.

In the probability of use survey, participants rated the probability of encountering a given sentence on a 7-point Likert scale (1 –*very unlikely*, 7 –*very likely*). Finally, the stereotypicality survey was divided into two parts, administered in a random order. Participants were asked to rate on a 7-point Likert scale the congruency of sentences with stereotypes assigned to females in one part and to males in another part (1 –*very incongruent*, 7 –*very congruent*), aiming to thoroughly assess both feminine and masculine stereotypical values associated with each sentence. The two separate subscales, presented in a counterbalanced order, allowed us to account for potential priming effects and thus to enhance the robustness and validity of our results by addressing potential biases introduced by the order of presentation.

Having completed a survey, participants provided basic demographic information, including their age, gender, and the highest level of education. Finally, participants completed the Polish version of the Bem Sex Role Inventory (BSRI [27], adapted into Polish by Lipińska-Grobelny & Gorczycka [28]), in which they rated on a 5-point Likert scale how much each of the 20 characteristics (10 feminine, 10 masculine) corresponded to them (1 –*I am definitely not like that*, 5 –*I am definitely like that*). The BSRI aims to assess feminine and masculine characteristics, which helps to determine the gender role with which a participant identifies.

#### 2.1.4. Data analysis

*2*.*1*.*4*.*1*. *Reliability of the ratings*. Inter-class correlation coefficients (ICC) were calculated using a two-way random-effect model based on the mean of multiple raters as a measure of inter-rater reliability. Also, Cronbach’s alpha (*α*) coefficients were calculated to assess internal consistency of the meaningfulness, probability of use, and stereotypicality scales across all surveys.

*2*.*1*.*4*.*2*. *Sentence types*. The meaningfulness ratings were analyzed using linear mixed-effects models (LMMs [29–32]) with Sentence type (Semantically correct vs. Semantically incorrect vs. Stereotypically congruent vs. Stereotypically incongruent) as a within-subject factor, using the *lme4* package [33] for R [34]. In contrast, the probability of use and stereotypicality ratings were also analyzed using LMMs (see above) with Sentence type (Semantically correct vs. Stereotypically congruent vs. Stereotypically incongruent) as a within-subject factor. The meaningfulness, probability of use, and stereotypicality ratings were analyzed using LMMs, which allowed for the assessment of potential differences across the respective conditions, additionally accounting for participant- and item-related variability. Specifically, a maximal model was first computed with a full random-effect structure, including participant-related and item-related variance components for intercepts and by-participant and by-item random slopes for fixed effects [31]. When the data did not support the execution of the maximal model random structure, we reduced the model complexity to arrive at a parsimonious model [35]. To do so, we computed principal component analyses of the random structure and then kept the number of principal components that cumulatively accounted for 100% of the variance. *b* estimates and significance of fixed effects and interactions (*p*-values) were based on the Satterthwaite approximation for LMM (the *lmerTest* package [36]) for R [34]. *Post-hoc* analyses were calculated using the *emmeans* package [37] for R [34]. Violin plots were used to visualize the results, as they combine the benefits of box plots and kernel density plots, allowing for a more detailed visualization of the data distribution.

*2*.*1*.*4*.*3*. *Stereotypicality and gender*. To explore the role of gender in the perception of stereotype-related sentences, the meaningfulness, probability of use, and stereotypicality ratings were analyzed separately using LMMs (see above) on a basis of 2 (Gender: Females vs. Males) × 2 (Sentence type: Stereotypically congruent vs. Stereotypically incongruent) between-subject design.

*2*.*1*.*4*.*4*. *Stereotypicality and gender schemas*. *To* further explore whether gender schemas affect the perception of stereotype-related sentences, the meaningfulness, probability of use, and stereotypicality ratings we analyzed separately using LMMs (see above) on a basis of 4 (Gender schema: Sex-typed vs. Cross-sex–typed vs. Androgynous vs. Undifferentiated) × 2 (Sentence type: Stereotypically congruent vs. Stereotypically incongruent) between-subject design.

Participants were categorized into four gender schemas based on the results of the BSRI [28] following Bem [23]. Initially, individual ratings of feminine and masculine characteristics from the BSRI [28] were summed up to calculate participants’ overall femininity and masculinity scores within the 0–50 range. Subsequently, employing a median split of femininity and masculinity scores, participants were assigned to four categories: sex-typed, cross-sex–typed, androgynous, and undifferentiated individuals (see Table 1 below for details).

**Table 1:** Distribution of the number of participants classified into respective gender schemas across the meaningfulness, probability of use, and stereotypicality ratings in Polish and English (*N*_*Polish*_ = 472; *N*_*English*_ = 470).

	Meaningfulness	Probability of use	Stereotypicality
			**Polish**
*Sex-typed*	51	28	54
*Cross-sex–typed*	45	36	33
*Androgynous*	40	31	20
*Undifferentiated*	41	53	40
*=*	177	148	147
	**English**		
*Sex-typed*	45	42	38
*Cross-sex–typed*	36	19	27
*Androgynous*	45	36	43
*Undifferentiated*	50	51	38
*=*	176	148	146

Sex-typed individuals–women: femininity score > = 39, masculinity score <34; men: femininity score <38, masculinity score > = 35; Cross-sex–typed individuals–women: femininity scale <38, masculinity score > = 35; men: femininity score > = 39, masculinity score <34; Androgynous individuals–women and men: femininity scores > = 39, masculinity score > = 35; Undifferentiated individuals–women and men: femininity score <38, masculinity score <34.

### 2.2. Results

#### 2.2.1. Reliability of the ratings

ICCs pointed to very high inter-rater reliability for all the meaningfulness (ICC = .99), probability of use (ICC = .92), and stereotypicality (ICC = .98) ratings of Polish sentences. Also, the meaningfulness (*α* = .88), probability of use (*α* = .92), and stereotypicality (*α* = .89) scales demonstrated high degrees of internal consistency across all Polish surveys.

#### 2.2.2. Sentence types

**Fig 1:**
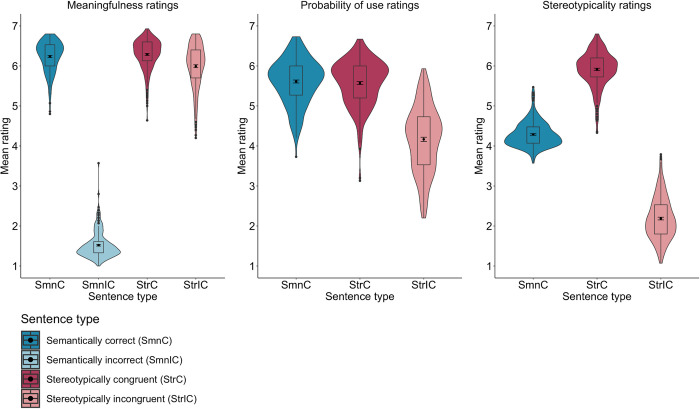
Mean meaningfulness, probability of use, and stereotypicality ratings of Polish semantically correct and -incorrect as well as stereotypically congruent and -incongruent sentences (*N* = 472).

*2*.*2*.*2*.*1*. *Meaningfulness ratings*. The analysis of the meaningfulness ratings of Polish sentences revealed a fixed effect of Sentence type, whereby semantically incorrect sentences were rated as the least meaningful compared to semantically correct, *b* = –4.72, SE = .04, *t*(471.6) = –106.44, *p* < .001, stereotypically congruent, *b* = –4.80, SE = 04, *t*(480.7) = –105.44, *p* < .001, and stereotypically incongruent sentences, *b* = –4.52, SE = 04, *t*(481.4) = –99.13, *p* < .001. Then, stereotypically incongruent sentences were rated as less meaningful than both semantically correct, *b* = .20, SE = 04, *t*(479.3) = 4.47, *p* < .001, and stereotypically congruent sentences, *b* = .28, SE = 04, *t*(472.2) = 6.38, *p* < .001. There was no statistically significant difference between semantically correct and stereotypically congruent sentences, *b* = –.08, SE = 04, *t*(478.7) = –1.79, *p* = .073 (see **Table 2** and **Fig 1A**).

**Table 2:** Mean meaningfulness, probability of use, and stereotypicality ratings (with 95% confidence intervals) of Polish semantically correct and incorrect as well as stereotypically congruent and incongruent sentences (*N* = 472).

Sentence types	Meaningfulness	Probability of use	Stereotypicality
*Stereotypically congruent*	6.29 [6.19, 6.39]	5.56 [5.41, 5.70]	5.92 [5.81, 6.03]
*Stereotypically incongruent*	6.00 [5.90, 6.10]	4.17 [3.97, 4.37]	2.18 [2.05, 2.31]
*Semantically correct*	6.20 [6.10, 6.31]	5.60 [5.46, 5.75]	4.32 [4.19, 4.44]
*Semantically incorrect*	1.48 [1.37, 1.58]	–	–

*2*.*2*.*2*.*1*. *Probability of use ratings*. The analysis of the probability of use ratings of Polish sentences showed a fixed effect of Sentence type, such that stereotypically incongruent sentences were rated as less probable to be used in everyday contexts than both semantically correct, *b* = 1.43, SE = .10, *t*(314.4) = 13.32, *p* < .001, and stereotypically congruent sentences, *b* = 1.38, SE = .10, *t*(304.1) = 12.68, *p* < .001. Semantically correct and stereotypically congruent sentences were rated as equally probable to be used in everyday contexts, *b* = .04, SE = .07, *t*(354.8) = .65, *p* = .514 (see **Table 2** and **Fig 1B**).

*2*.*2*.*2*.*3*. *Stereotypicality ratings*. The analysis of the stereotypicality ratings of Polish sentences revealed a fixed effect of Sentence type, whereby stereotypically congruent sentences were rated as more stereotypical than semantically correct, *b* = –1.59, SE = .07, *t*(258.9) = –20.97, *p* < .001, and stereotypically incongruent sentences, *b* = 3.73, SE = .10, *t*(216.1) = 36.94, *p* < .001. Then, semantically correct sentences were rated as more stereotypical than stereotypically incongruent sentences, *b* = 2.13, SE = .08, *t*(235.5) = 25.05, *p* < .001 (see **Table 2** and **Fig 1C**).

#### 2.2.3. Stereotypicality and gender

*2*.*2*.*3*.*1*. *Meaningfulness ratings*. The analysis of the meaningfulness ratings of Polish sentences revealed a fixed effect of Sentence type, with higher meaningfulness ratings of stereotypically congruent than incongruent sentences, *b* = .29, SE = .06, *t*(313.0) = 4.87, *p <* .001. There was also a fixed effect of Gender, whereby females rated sentences as more meaningful than males, *b* = .33, SE = .10, *t*(173.7) = 3.26, *p* = .001.

**Fig 2:**
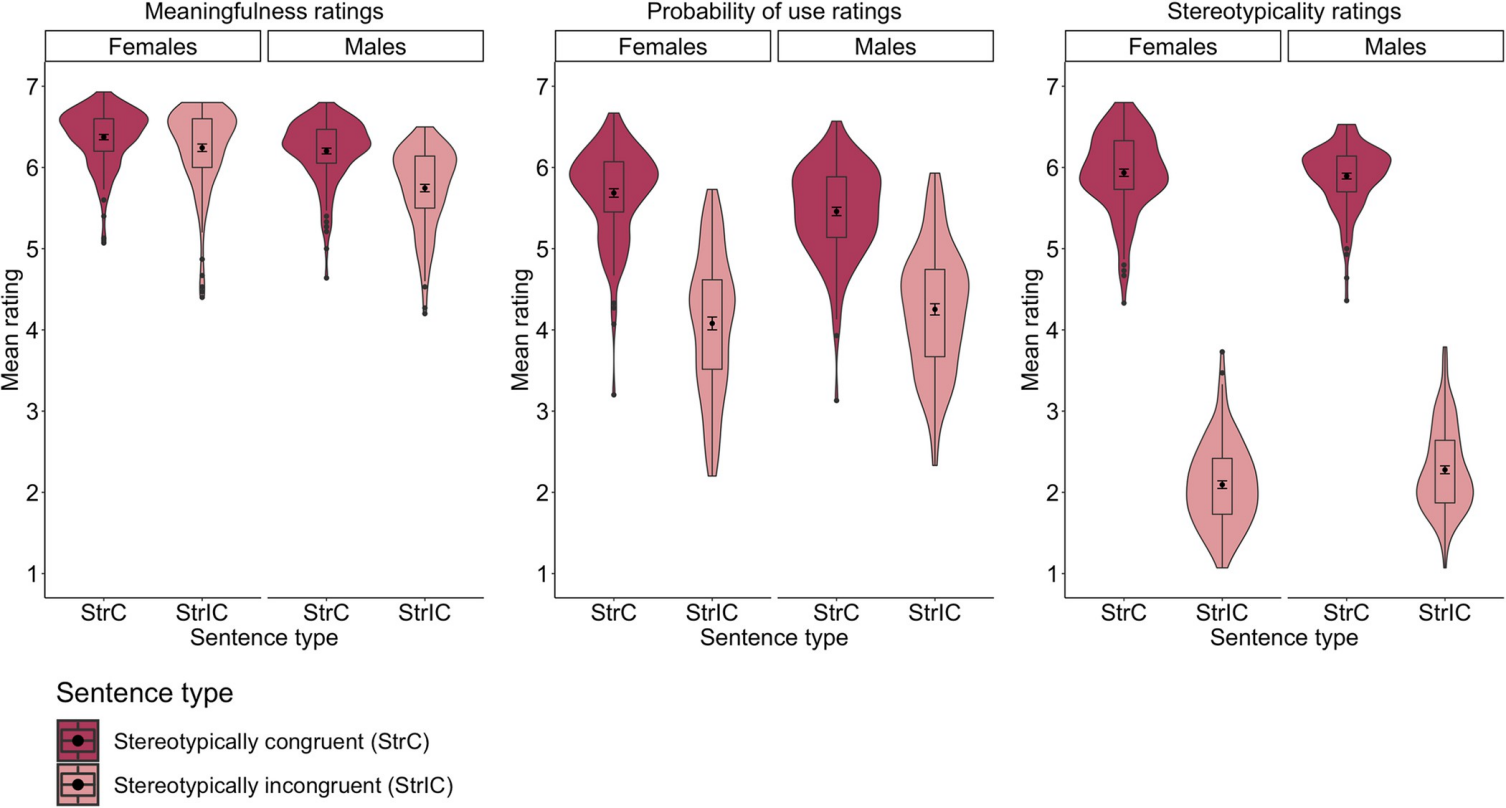
Mean meaningfulness, probability of use, and stereotypicality ratings of polish stereotypically congruent and incongruent sentences grouped by participants’ gender (*N*_*Females*_ = 240; *N*_*Males*_ = 232).

The analysis also revealed a Sentence type × Gender interaction. *Post-hoc* comparisons showed that males rated stereotypically congruent sentences as more meaningful than stereotypically incongruent sentences, *b* = .45, SE = .07, *t*(283.9) = 6.23, *p* < .001, whereas females rated the two sentence types as similarly meaningful, *b* = .12, SE = .06, *t*(274.5) = 1.80, *p* = .072. Moreover, females rated stereotypically incongruent sentences as more meaningful than males, *b* = .50, SE = .12, *t*(173.0) = 4.05, *p* < .001, and there was no such a between-gender difference for stereotypically congruent sentences, *b* = .17, SE = .09, *t*(171.9) = 1.82, *p =* .070 (see **Table 3** and **Fig 2A**).

**Table 3:** Mean meaningfulness, probability of use, and stereotypicality ratings (with 95% confidence intervals) of Polish stereotypically congruent and incongruent sentences, grouped by participants’ gender (*N*_*Females*_ = 240; *N*_*Males*_ = 232).

Sentence types	Gender	Meaningfulness	Probability of use	Stereotypicality
Stereotypically congruent	*Females*	6.37 [6.23, 6.52]	5.67 [5.48, 5.86]	5.93 [5.78, 6.09]
	*Males*	6.20 [6.05, 6.34]	5.45 [5.26, 5.63]	5.90 [5.75, 6.05]
Stereotypically incongruent	*Females*	6.25 [6.07, 6.43]	4.07 [3.80, 4.34]	2.09 [1.91, 2.26]
	*Males*	5.74 [5.56, 5.93]	4.27 [4.01, 4.54]	2.28 [2.10, 2.45]

*2*.*2*.*3*.*2*. *Probability of use ratings*. The analysis of the probability of use ratings of Polish sentences showed a fixed effect of Sentence type, with higher probability of use ratings for stereotypically congruent than incongruent sentences, *b* = 1.38, SE = .11, *t*(298.4) = 12.34, *p <* .001. However, there was no statistically significant fixed effect of Gender, *b* = .01, SE = .12, *t*(145.2) = .08, *p* = .936.

The analysis also showed a Sentence type × Gender interaction. *Post-hoc* comparisons revealed a trend pointing to higher probability of use ratings for stereotypically congruent sentences in females than in males, *b* = .22, SE = .11, *t*(148.4) = 1.88, *p* = .061, and there was no between-gender difference in the probability ratings of stereotypically incongruent sentences, *b* = –.20, SE = .17, *t*(145.3) = –1.11, *p* = .265 (see **Table 3** and **Fig 2B**).

*2*.*2*.*3*.*3*. *Stereotypicality ratings*. The analysis of the stereotypicality ratings of Polish sentences showed a fixed effect of Sentence type, with higher stereotypicality ratings for stereotypically congruent than incongruent sentences, *b* = 3.73, SE = .10, *t*(232.6) = 35.71, *p <* .001. However, the fixed effect of Gender, *b* = –.07, SE = .05, *t*(155.6) = –1.45, *p* = .147, as well as a Sentence type × Gender interaction, *b* = .22, SE = .18, *t*(149.5) = 1.24, *p* = .216, were not statistically significant (see **Table 3** and **Fig 2C**).

#### 2.2.4. Stereotypicality and gender role identities

*2*.*2*.*4*.*1*. *Meaningfulness ratings*. The analysis of the meaningfulness ratings of Polish sentences revealed a fixed effect of Sentence type, with higher meaningfulness ratings for stereotypically congruent than incongruent sentences, *b* = .28, SE = .06, *t*(314.0) = 4.75, *p* < .001. However, there was no statistically significant fixed effect of Gender role, *b* = –.09, SE = .15, *t*(174.9) = –.64, *p* = .517.

**Fig 3:**
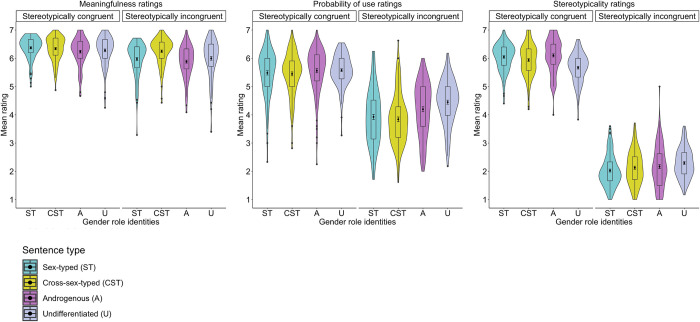
Mean meaningfulness (A), probability of use (B), and stereotypicality (C) ratings of Polish semantically correct and incorrect as well as stereotypically congruent and incongruent sentences grouped by participants’ gender role identities (*N*_*Sex-typed*_ = 133; *N*_*Cross-sex–typed*_ = 114; *N*_*Androgynous*_ = 91; *N*_*Undifferentiated*_ = 134).

The analysis also revealed a Sentence type × Gender role interaction. First, *post-hoc* comparisons showed that stereotypically congruent sentences were rated as more meaningful than stereotypically incongruent sentences by sex-typed, *b* = .41, SE = .08, *t*(276.6) = 4.79, *p* < .001, androgenous, *b* = .39, SE = .09, *t*(258.9) = 4.15, *p* < .001, as well as undifferentiated individuals, *b* = .25, SE = .09, *t*(259.6) = 2.75, *p* = .006; however, stereotypically congruent and incongruent sentences were rated as similarly meaningful by cross-sex–typed individuals, *b* = .06, SE = .09, *t*(265.7) = –.11, *p =* .481. Second, stereotypically incongruent sentences were rated as more meaningful by cross-sex–typed individuals than by sex-typed, *b* = –.35, SE = .17, *t*(173.6) = –2.05, *p* = .040, and androgenous individuals, *b* = –.44, SE = .18, *t*(172.8) = –2.41, *p* = .016; however, stereotypically incongruent sentences were also rated as similarly meaningful by cross-sex–typed and undifferentiated individuals, *b* = .24, SE = .18, *t*(172.3) = 1.34, *p* = .181(see **Table 4** and **Fig 3A**).

**Table 4:** Mean meaningfulness, probability of use, and stereotypicality ratings (with 95% confidence intervals) of Polish stereotypically congruent and incongruent sentences, grouped by participants’ gender role identities (*N*_*Sex-typed*_ = 133; *N*_*Cross-sex–typed*_ = 114; *N*_*Androgynous*_ = 91; *N*_*Undifferentiated*_ = 134).

	Gender role identities			
	*Sex-typed*	*Cross-sex–typed*	*Androgenous*	*Undifferentiated*
**Meaningfulness**				
*Stereotypically congruent sentences*	6.32 [6.13, 6.51]	6.32 [6.13, 6.52]	6.21 [6.00, 6.42]	6.27 [6.06, 6.48]
*Stereotypically incongruent sentences*	5.90 [5.66, 6.14]	6.26 [6.00, 6.52]	5.81 [5.54, 6.09]	6.01 [5.75, 6.28]
**Probability of use**				
*Stereotypically congruent sentences*	5.53 [5.24, 5.82]	5.46 [5.20, 5.71]	5.62 [5.35, 5.89]	5.61 [5.39, 5.83]
*Stereotypically incongruent sentences*	3.80 [3.40, 4.21]	3.87 [3.51, 4.23]	4.28 [3.89, 4.67]	4.50 [4.20, 4.80]
**Stereotypicality**				
*Stereotypically congruent sentences*	6.05 [5.88, 6.21]	5.88 [5.67, 6.08]	6.09 [5.83, 6.35]	5.69 [5.50, 5.88]
*Stereotypically incongruent sentences*	2.03 [1.83, 2.23]	2.17 [1.92, 2.41]	2.34 [2.03, 2.65]	2.30 [2.08, 2.53]

*2*.*2*.*4*.*2*. *Probability of use ratings*. The analysis of the probability of use ratings of Polish sentences revealed a fixed effect of Sentence type, with higher probability of use ratings for stereotypically congruent than incongruent sentences, *b* = 1.44, SE = .11, *t*(291.6) = 12.60, *p* < .001. There was also a fixed effect of Gender role, whereby undifferentiated individuals rated Polish sentences as more probable to be used in everyday contexts than both sex-typed, *b* = –.38, SE = .17, *t*(142.1) = –2.23, *p* = .026, and cross-sex–typed individuals, *b* = –.39, SE = .15, *t*(141.6) = –2.43, *p* = .016. Yet, there was no difference in the probability of use ratings between undifferentiated and androgenous individuals, *b* = –.10, SE = .16, *t*(141.6) = –.61, *p* = .540.

The analysis also revealed a Sentence type × Gender role interaction. *Post-hoc* comparisons showed that undifferentiated individuals rated stereotypically incongruent sentences as more probable to be used in everyday contexts than sex-typed, *b* = –.69, SE = .24, *t*(142.2) = –2.81, *p* < .001, and cross-sex–typed individuals, *b* = –.62, SE = .22, *t*(141.5) = –2.74, *p* = .006; however, stereotypically incongruent sentences were rated as equally probable to be used in everyday contexts by androgenous and undifferentiated individuals, *b* = –.22, SE = .23, *t*(141.9) = –.92, *p* = .358 (see **Table 4** and **Fig 3B**).

*2*.*2*.*4*.*3*. *Stereotypicality ratings*. The analysis of the stereotypicality ratings of Polish sentences revealed a fixed effect of Sentence type, with higher stereotypicality ratings for stereotypically congruent than incongruent sentences, *b* = 3.71, SE = .11, *t*(221.8) = 34.24, *p <* .001. There was also a fixed effect of Gender role, whereby androgenous individuals rated Polish sentences as more stereotypical than sex-typed, *b* = –.17, SE = .08, *t*(146.2) = –2.14, *p* = .033, cross-sex–typed, *b* = .19, SE = .08, *t*(142.5) = 2.17, *p* = .031, and undifferentiated individuals, *b* = .22, SE = .08, *t*(141.9) = 2.58, *p* = .010.

The analysis also revealed a Sentence type × Gender role interaction. *Post-hoc* comparisons showed that undifferentiated individuals rated stereotypically congruent sentences as less stereotypical than sex-typed, *b* = .35, SE = .11, *t*(145.0) = 3.04, *p* = .002, and androgenous individuals, *b* = .40, SE = .15, *t*(143.5) = 2.62, *p* = .009; however, stereotypically congruent sentences were rated as similarly stereotypical by cross-sex–typed and undifferentiated individuals, *b* = .19, SE = .13, *t*(142.9) = 1.44, *p* = .151(see **Table 4** and **Fig 3C**).

### 2.3. Discussion

The series of survey studies on Polish sentences aimed to test the degree of gender stereotype load ingrained in sentences that we designed as either in line with or violating stereotypes associated with males or females. To this end, participants assessed gender stereotypically congruent and incongruent sentences, along with semantically correct and incorrect sentences (i.e., control conditions), on the meaningfulness, probability of use, and stereotypicality scales.

First of all, all the sentences were assessed in line with the categories ascribed to them. Namely, stereotypically congruent relative to semantically correct sentences were rated as both comparably meaningful as well as probable to be used in everyday contexts, with higher stereotypicality ratings for stereotypically congruent sentences. Predictably, such a pattern indicates that both sentence types were evaluated as equally meaningful and probable, and they differed only in terms of their gender stereotypicality. Then, stereotypically incongruent sentences were evaluated as somewhat less meaningful and much less probable to be used in everyday context than stereotypically congruent and semantically correct sentences, at the same time receiving the lowest stereotypicality ratings. Such results suggest that stereotypically incongruent sentences were expectedly evaluated as strongly violating widespread gender stereotypes and, therefore, less probable in everyday communicative interactions. Note that stereotypically incongruent sentences, receiving significantly higher ratings on the meaningfulness scale than semantically incorrect sentences, were assessed as adhering to conceptual knowledge. This inference allows us to conclude that the observed effect on the stereotype congruency scale was driven by the violation of stereotype-based knowledge. Altogether, this confirms that the four sentence types included in our database are well-selected to illustrate the how congruency with gender stereotypes modulates the evaluation of different sentence types in terms of their meaningfulness and probability of use. At the same time, the results pointed to a critical role of participants’ gender and gender roles in how they evaluated the presented sentences. First, males assessed stereotypically incongruent sentences as less meaningful than stereotypically congruent sentences, with no such differences observed among female participants. Second, females rated stereotypically congruent sentences as more probable to be used in everyday language compared to males, which additionally points to the role of gender in how linguistic information that either confirms or violates gender expectations is evaluated. Furthermore, we observed more stereotype-driven evaluations of stereotypically incongruent sentences given by sex-typed individuals.

The aim of Study II was to further test whether and to what extent the effects observed for Polish items were replicated for English sentences.

## 3. Study II: The perception of English sentences

### 3.1. Method

#### 3.1.1. Participants

Overall, 510 native speakers of English (258 females, 252 males) took part in Study II. All of them resided in an English-speaking country when taking the survey. Following the exclusion of 40 participants (7.84%; 18 females, 22 males) due to providing incomplete answers, the final sample included 470 respondents (240 females, 230 males), distributed across three surveys: the meaningfulness (176 participants; 90 females, 86 males), probability of use (148 participants; 75 females, 73 males), and stereotypicality surveys (146 participants; 75 females, 71 males). Similar to Study I, participants’ ages ranged from 18 to 40 years old (*M* = 27.34 years, SD = 5.54; meaningfulness: *M* = 27.48, *SD* = 5.59; probability of use: *M* = 27.59, *SD* = 5.80; stereotypicality: *M* = 26.90, *SD* = 5.22). As for participants’ education, their largest number held a bachelor’s degree (41.70%) and a master’s degree), with less than one fifth having graduated from high school (18.72%).

#### 3.1.2. Stimuli

The stimuli for Study II were designed analogously to those in Study I. The sentences were controlled for length (*M* = 9.0; range: 8–10), and included a stereotypically feminine (*n* = 60 per sentence type) or masculine (*n* = 60 per sentence type) critical noun placed in a mid-sentence position (i.e., always as the fourth word from the end). Similar to Study I, the critical nouns included stereotypical gender nouns in Polish and English. The critical nouns were all matched on length, the number of letters and syllables, frequency (SUBTLEX-UK [38]), valence and arousal [39], concreteness, and age of acquisition [40].

#### 3.1.3. Procedure

The procedure was the same as in Study I, with the exception of the language in which the sentences were constructed (i.e., Polish in Study I and English in Study II).

#### 3.1.4. Data analysis

Data analyses for English sentences were performed in line with those run for Polish sentences (see section 2.1.4. for details).

### 3.2. Results

#### 3.2.1. Reliability of the ratings

ICCs pointed to very high inter-rater reliability for all the meaningfulness (ICC = .98), probability of use (ICC = .87), and stereotypicality (ICC = .97) ratings of English sentences. Also, the meaningfulness (*α*_*=*_ .88), probability of use (*α*_*=*_ .89), and stereotypicality (*α*_*=*_ .88) scales demonstrated high degrees of internal consistency across all English surveys.

#### 3.2.2. Sentence types

**Fig 4:**
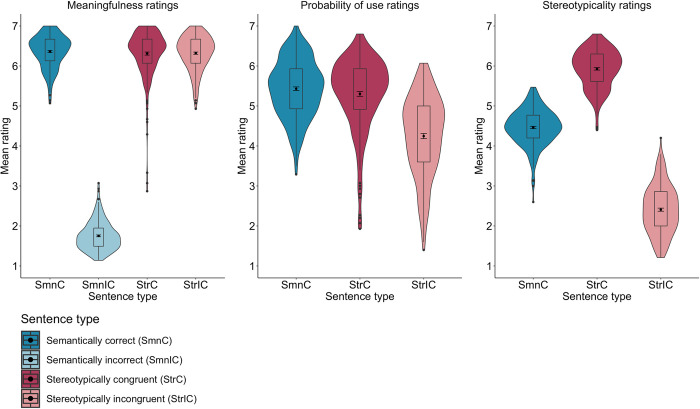
Mean meaningfulness, probability of use, and stereotypicality ratings of English semantically correct and incorrect as well as stereotypically congruent and incongruent sentences (*N* = 470).

*3*.*2*.*2*.*1*. *Meaningfulness ratings*. The analysis of the meaningfulness ratings of English sentences revealed a fixed effect of Sentence type, whereby semantically incorrect sentences were rated as the least meaningful compared to semantically correct, *b* = 4.70, SE = .04, *t*(475.0) = 99.13, *p* < .001, stereotypically congruent, *b* = –4.64, SE = .04, *t*(473.3) = –98.50, *p* < .001, and stereotypically incongruent sentences, *b* = –4.64, SE = .04, *t*(473.5) = –98.44, *p* < .001. Then, there were no differences in meaningfulness between semantically correct and stereotypically congruent sentences, *b* = .06, SE = .04, *t*(469.2) = 1.32, *p* = .185, as well as semantically correct and stereotypically incongruent sentences, *b* = .05, SE = .04, *t*(469.2) = 1.28, *p* < .001. Finally, there was also no difference in meaningfulness between stereotypically congruent and stereotypically incongruent sentences, *b* <–.01, SE = .04, *t*(469.2) = –.04, *p* = .967 (see **Table 5** and **Fig 4A**).

**Table 5:** Mean meaningfulness, probability of use, and stereotypicality ratings (with 95% confidence intervals) of English semantically correct and incorrect as well as stereotypically congruent and incongruent sentences (*N* = 470).

Sentence types	Meaningfulness	Probability of use	Stereotypicality
*Stereotypically congruent*	6.30 [6.19, 6.41]	5.25 [5.06, 5.45]	5.88 [5.75, 6.01]
*Stereotypically incongruent*	6.30 [6.19, 6.42]	4.20 [3.97, 4.44]	2.46 [2.29, 2.63]
*Semantically correct*	6.36 [6.25, 6.48]	5.41 [5.22, 5.60]	4.45 [4.34, 4. 57]
*Semantically incorrect*	1.66 [1.54, 1.77]	–	–

*3*.*2*.*2*.*2*. *Probability of use ratings*. The analysis of the probability of use ratings of English sentences showed a fixed effect of Sentence type, such that stereotypically incongruent sentences were rated as less probable to be used in everyday contexts than both semantically correct, *b* = 1.20, SE = .12, *t*(357.7) = 9.28, *p* < .001, and stereotypically congruent sentences, *b* = 1.05, SE = .12, *t*(357.9) = 8.11, *p* < .001. Yet, there was no difference in probability of use ratings of semantically correct and stereotypically congruent sentences, *b* = .15, SE = .09, *t*(356.6) = 1.59, *p* = .110 (see **Table 5** and **Fig 4B**).

*3*.*2*.*2*.*3*. *Stereotypicality ratings*. The analysis of the stereotypicality ratings of English sentences revealed a fixed effect of Sentence type, whereby stereotypically congruent sentences were rated as more stereotypical than semantically correct, *b* = –1.42, SE = .08, *t*(248.7) = –16.47, *p* < .001, and stereotypically incongruent sentences, *b* = 3.42, SE = .13, *t*(183.6) = 26.06, *p* < .001. Then, semantically correct sentences were rated as more stereotypical than stereotypically incongruent sentences, *b* = 1.99, SE = .09, *t*(231.5) = 21.10, *p* < .001 (see **Table 5** and **Fig 4C**).

#### 3.2.3. Stereotypicality and gender

**Fig 5:**
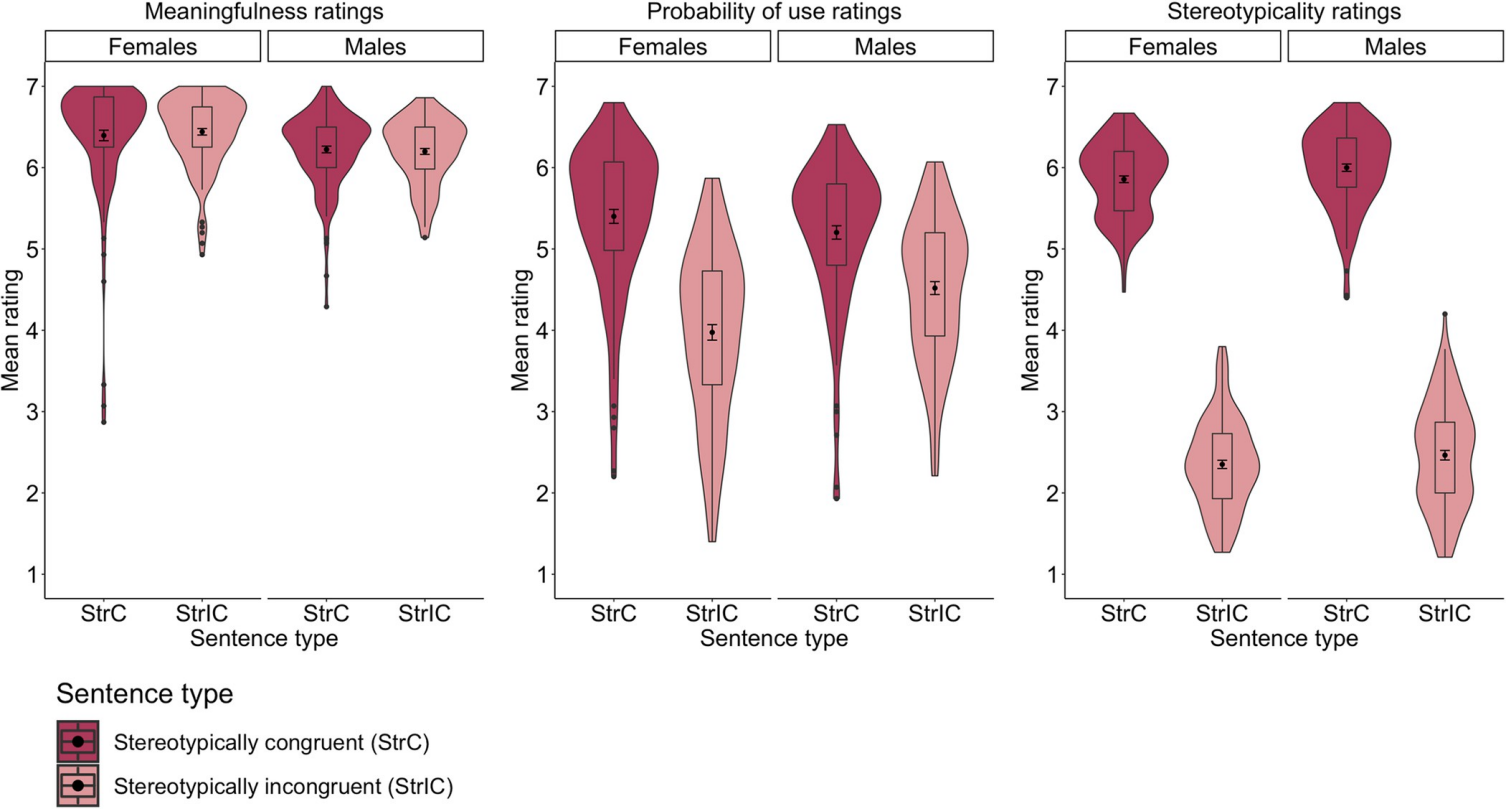
Mean meaningfulness, probability of use, and stereotypicality ratings of English stereotypically congruent and incongruent sentences grouped by participants’ gender (*N*_*Females*_ = 240; *N*_*Males*_ = 230).

*3*.*2*.*3*.*1*. *Meaningfulness ratings*. The analysis of the meaningfulness ratings of English sentences did not reveal a statistically significant effect of Sentence type, *b* < .01, SE = .05, *t*(240.4) < .01, *p* = .993, Gender, *b* = .19, SE = .10, *t*(170.5) = 1.78, *p* = .076, or a Sentence type × Gender interaction, *b* = –.05, SE = .05, *t*(151.9) = –1.06, *p* = .290 (see **Table 6** and **Fig 5A**).

**Table 6:** Mean meaningfulness, probability of use, and stereotypicality ratings (with 95% confidence intervals) of English stereotypically congruent and incongruent sentences, grouped by participants’ gender (*N*_*Females*_ = 240; *N*_*Males*_ = 230).

Sentence types	Gender	Meaningfulness	Probability of use	Stereotypicality
Stereotypically congruent	*Females*	6.38 [6.22, 6.54]	5.39 [5.12, 5.65]	5.84 [5.67, 6.02]
	*Males*	6.21 [6.06, 6.37]	5.11 [4.87, 5.36]	5.93 [5.75, 6.10]
Stereotypically incongruent	*Females*	6.41 [6.23, 6.58]	3.96 [3.65, 4.28]	2.36 [2.13, 2.58]
	*Males*	6.19 [6.01, 6.36]	4.44 [4.14, 4.75]	2.57 [2.35, 2.80]

*3*.*2*.*3*.*2*. *Probability of use ratings*. The analysis of the probability of use ratings of English sentences showed fixed effect of Sentence type, *b* = 1.04, SE = .13, *t*(325.7) = 7.87, *p* < .001. However, there was no statistically significant fixed effect of Gender, *b* = –.10, SE = .15, *t*(141.2) = –.65, *p* = .511.

The analysis also revealed a Sentence type × Gender interaction, *b* = .75, SE = .18, *t*(147.8) = 4.16, *p* < .001. *Post-hoc* comparisons showed that while males rated stereotypically incongruent sentences as more probable to be used in everyday contexts than females, *b* = –.47, SE = .20, *t*(144.5) = –2.38, *p =* .018, there was no between-gender difference in probability ratings for stereotypically congruent sentences, *b* = .27, SE = .15, *t*(137.7) = 1.74, *p* = .082 (see **Table 6** and **Fig 5B**).

*3*.*2*.*3*.*3*. *Stereotypicality ratings*. The analysis of the stereotypicality ratings of English sentences revealed a fixed effect of Sentence type, with higher stereotypicality ratings of stereotypically congruent than incongruent sentences, *b* = 3.42, SE = .13, *t*(185.7) = 25.79, *p <* .001. There was also a fixed effect of Gender, such that males rated English sentences as more stereotypical than females, *b* = –.14, SE = .06, *t*(134.2) = –2.34, *p* = .020. However, the analysis showed no statistically significant Sentence type × Gender interaction, *b* = .13, SE = .24, *t*(140.2) = .54, *p* = .584 (see **Table 6** and **Fig 5C**).

#### 3.2.4. Stereotypicality and gender schemas

**Fig 6:**
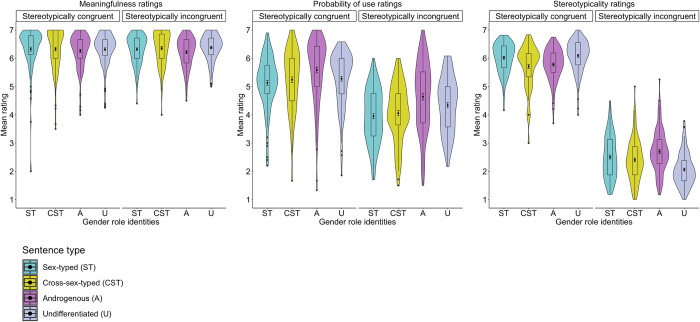
Mean meaningfulness, probability of use, and stereotypicality ratings of English semantically correct and incorrect as well as stereotypically congruent and incongruent sentences grouped by participants’ gender role identities (*N*_*Sex-typed*_ = 125; *N*_*Cross-sex–typed*_ = 82; *N*_*Androgynous*_ = 124; *N*_*Undifferentiated*_ = 139).

*3*.*2*.*4*.*1*. *Meaningfulness ratings*. The analysis of the meaningfulness ratings of English sentences revealed no statistically significant fixed effect of Sentence type, *b* = .01, SE = .07, *t*(328.9) = .16, *p* = .876, Gender schema, *b* = –.15, SE = .16, *t*(167.9) = –.95, *p* = .341. There was also no statistically significant Sentence type × Gender schema interaction, *b* = –.02, SE = .07, *t*(166.8) = –.31, *p* = .754 (see **Table 7** and **Fig 6A**).

**Table 7:** Mean meaningfulness, probability of use, and stereotypicality ratings (with 95% confidence intervals) of English stereotypically congruent and incongruent sentences, grouped by participants’ gender schemas (*N*_*Sex-typed*_ = 125; *N*_*Cross-sex–typed*_ = 82; *N*_*Androgynous*_ = 124; *N*_*Undifferentiated*_ = 139).

	Gender schemas			
	*Sex-typed*	*Cross-sex–typed*	*Androgenous*	*Undifferentiated*
**Meaningfulness**				
*Stereotypically congruent sentences*	6.39 [6.17, 6.61]	6.23 [5.98, 6.47]	6.29 [6.07, 6.51]	6.28 [6.07, 6.48]
*Stereotypically incongruent sentences*	6.38 [6.15, 6.62]	6.24 [5.98, 6.50]	6.24 [6.00, 6.47]	6.32 [6.10, 6.55]
**Probability of use**				
*Stereotypically congruent sentences*	5.09 [4.78, 5.41]	5.15 [4.70, 5.60]	5.53 [5.19, 5.86]	5.23 [4.94, 5.52]
*Stereotypically incongruent sentences*	3.84 [3.44, 4.24]	4.08 [3.52, 4.65]	4.56 [4.14, 4.99]	4.29 [3.93, 4.65]
**Stereotypicality**				
*Stereotypically congruent sentences*	6.05 [5.82, 6.28]	5.83 [5.55, 6.10]	5.64 [5.41, 5.86]	6.03 [5.80, 6.27]
*Stereotypically incongruent sentences*	2.49 [2.19, 2.78]	2.38 [2.03, 2.73]	2.80 [2.52, 3.09]	2.11 [1.81, 2.41]

*3*.*2*.*4*.*2*. *Probability of use ratings*. The analysis of the probability of use ratings of English sentences revealed a fixed effect of Sentence type, with higher probability of use ratings for stereotypically congruent than incongruent sentences, *b* = 1.25, SE = .20, *t*(226.2) = 6.11, *p <* .001. There was also a fixed effect of Gender schema, such that androgenous individuals rated English sentences as more probable to be used in everyday contexts than sex-typed individuals, *b* = –.57, SE = .21, *t*(137.3) = –2.74, *p* = .006. All remaining differences were statistically non-significant, *p* > .05.

The analysis also revealed a Sentence type × Gender schema interaction. *Post-hoc* comparisons showed that stereotypically congruent sentences were rated as less probable to be used in everyday contexts by sex-typed than by androgenous individuals, *b* = –.43, SE = .21, *t*(131.9) = –2.00, *p* = .046; however, stereotypically congruent sentences were rated as similarly less probable to be used in everyday contexts by androgenous and cross-sex–typed individuals, *b* = .37, SE = .26, *t*(131.3) = 1.39, *p* = .164, as well as androgenous and undifferentiated individuals, *b* = .29, SE = .20, *t*(131.3) = 1.42, *p* = .157. In contrast, stereotypically incongruent sentences were rated as more probable to be used in everyday contexts by androgenous than sex-typed individuals, *b* = –.72, SE = .27, *t*(141.2) = –2.60, *p* = .010; however, stereotypically incongruent sentences were rated as similarly probable to be used in everyday contexts by androgenous and cross-sex–typed individuals, *b* = .48, SE = .34, *t*(141.0) = 1.38, *p* = .168, as well as androgenous and undifferentiated individuals, *b* = .27, SE = .26, *t*(140.9) = 1.02, *p* = .306. All remaining differences were statistically non-significant, *p* > .05 (see **Table 7** and **Fig 6B**).

*3*.*2*.*4*.*3*. *Stereotypicality ratings*. The analysis of the stereotypicality ratings of English sentences revealed a fixed effect of Sentence type, with higher stereotypicality ratings for stereotypically congruent than incongruent sentences, *b* = 3.44, SE = .13, *t*(184.5) = 26.40, *p <* .001. There was also a fixed effect of Gender schema, such that sex-typed individuals rated English sentences as more stereotypical than undifferentiated individuals, *b* = .19, SE = .08, *t*(132.3) = 2.21, *p* = .028. All remaining differences were statistically non-significant, *p* > .05.

The analysis also revealed a Sentence type × Gender schema interaction. *Post-hoc* comparisons showed that stereotypically congruent sentences were rated as less stereotypical by androgenous than sex-typed, *b* = .41, SE = .15, *t*(137.6) = 2.63, *p* = .009, and undifferentiated individuals, *b* = –.39, SE = .15, *t*(136.7) = –2.54, *p* = .120; however, stereotypically congruent sentences were rated as similarly stereotypical by androgenous and cross-sex–typed individuals, *b* = –.19, SE = .17, *t*(138.6) = –1.10, *p* = .270. Finally, stereotypically incongruent sentences were rated as more stereotypical by androgenous than undifferentiated individuals, *b* = .69, SE = .20, *t*(139.6) = 3.41, *p* < .001, and there was a trend towards higher stereotypicality ratings for androgenous than cross-sex–typed individuals, *b* = .42, SE = .22, *t*(140.6) = 1.88, *p* = .061; however, stereotypically incongruent sentences were rated as similarly stereotypical by androgenous and sex-typed individuals, *b* = –.31, SE = .20, *t*(140.2) = –1.56, *p* = .120. All remaining differences were statistically non-significant, *p* > .05 (see **Table 7** and **Fig 6C**).

### 3.3. Discussion

Similar to the results obtained in Study I, the survey studies conducted on English sentences showed that all the sentences were assessed in line with the categories ascribed to them. Namely, we replicated the results observed in Study I, whereby a graded effect of meaningfulness was observed, with semantically incorrect sentences assessed as the least meaningful, followed by stereotype incongruent sentence, and then stereotype congruent and semantically correct items. Also, stereotypically congruent and semantically correct sentences were comparably more probable to be used in everyday language compared to stereotypically incongruent sentences.

Interestingly, the analyses aimed to uncover the role of gender in the sentence ratings showed that while males in general evaluated the sentences as more stereotypical, they rated stereotype incongruent sentences as more probable to use compared to females. Finally, we observed more stereotype-driven evaluations of stereotypically incongruent sentences given by sex-typed individuals.

## 4. Between-language comparison: Polish vs. English

### 4.1. Method

#### 4.1.1. Data analysis

To explore potential language-specific differences between Polish and English, the meaningfulness ratings were analyzed separately using LMMs (see above) on a basis of 2 (Language: Polish vs. English) × 4 (Sentence type: Semantically correct vs. Semantically incorrect vs. Stereotypically congruent vs. Stereotypically incongruent) between-subject design. Then, the probability of use and stereotypicality ratings were analyzed using LMMs (see above) on a basis of 2 (Language: Polish vs. English) × 3 (Sentence type: Semantically correct vs. Semantically incorrect vs. Stereotypically congruent).

### 4.2. Results

#### 4.2.1. Meaningfulness ratings

The analysis of the meaningfulness ratings demonstrated a fixed effect of Sentence type, such that semantically incorrect sentences were rated as the least meaningful compared to semantically correct, *b* = 4.71, SE = .03, *t*(946.6) = 145.16, *p* < .001, stereotypically congruent, *b* = –4.73, SE = .03, *t*(953.0) = –144.01, *p* < .001, and stereotypically incongruent sentences, *b* = –4.58, SE = .03, *t*(953.9) = –138.6, *p* < .001. Then, stereotypically incongruent sentences were rated as less meaningful than semantically correct, *b* = .13, SE = .03, *t*(947.4) = 4.03, *p* < .001, and stereotypically incongruent sentences, *b* = .14, SE = .03, *t*(940.8) = 4.38, *p* < .001. Finally, there were no differences in meaningfulness between semantically correct and stereotypically congruent sentences, *b* = –.01, SE = .03, *t*(946.9) = –.03, *p* = .751. There was also a fixed effect of Language, such that English sentences were rated as more meaningful than Polish sentences, *b* = .16, SE = .06, *t*(413.9) = 2.47, *p* = .013.

**Fig 7:**
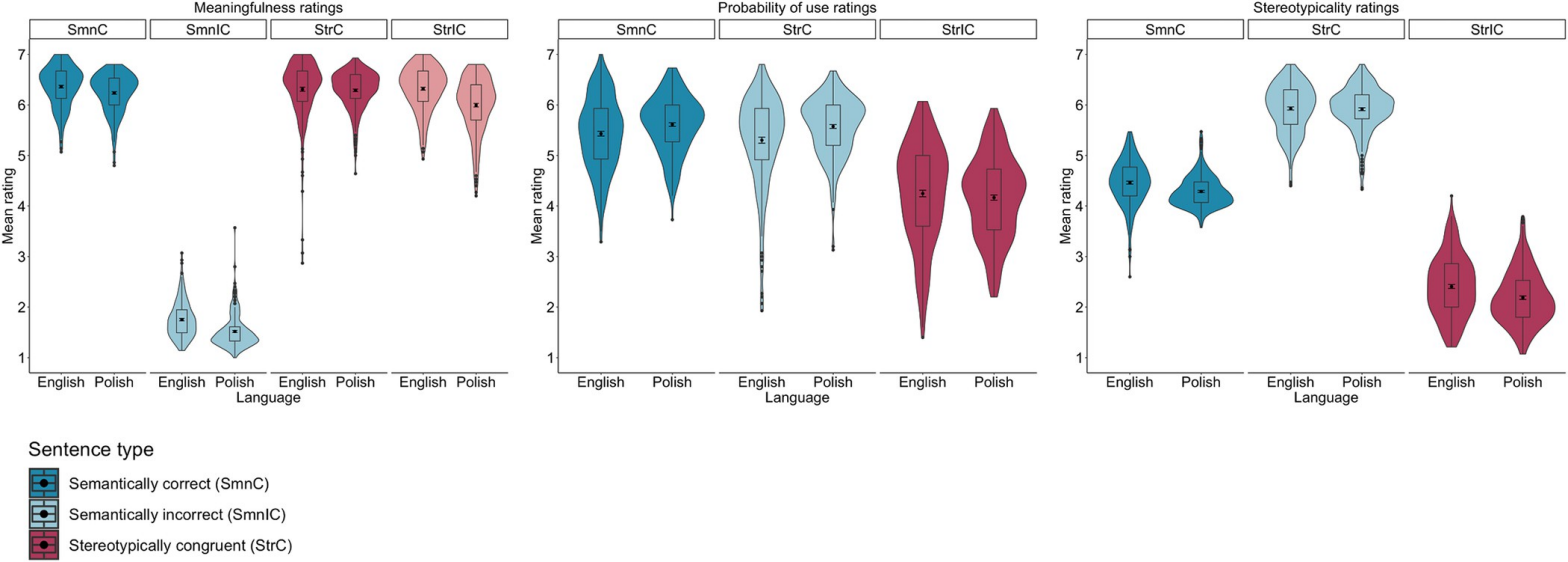
Mean meaningfulness, probability of use, and stereotypicality ratings of semantically correct and incorrect as well as stereotypically congruent and incongruent sentences grouped by language (Polish vs. English) (*N*_*Polish*_ = 472; *N*_*English*_ = 470).

The analysis also revealed a Sentence type × Language interaction. As for between-language comparisons, planned comparisons revealed that semantically correct, *b* = .15, SE = .07, *t*(699.2) = 2.04, *p* = .041, semantically incorrect, *b* = .18, SE = .07, *t*(715.8) = 2.32, *p* = .020, and stereotypically incongruent sentences, *b* = .30, SE = .07, *t*(688.6) = 3.90, *p* < .001, were rated as more meaningful in English than in Polish. However, stereotypically congruent sentences were rated as similarly meaningful in both languages, *b* = .01, SE = .07, *t*(688.5) = .19, *p* = .843 (see **Tables 2** and **5,** and **Fig 7**).

#### 4.2.2. Probability of use ratings

The analysis of the probability of use ratings showed a fixed effect of Sentence type, whereby stereotypically incongruent sentences were rated as less probable to be used in everyday contexts than semantically correct, *b* = 1.31, SE = .06, *t*(708.8) = 21.86, *p* < .001, and stereotypically congruent sentences, *b* = 1.22, SE = .06, *t*(700.9) = 20.44, *p* < .001. However, there was no statistically significant difference between semantically correct and stereotypically congruent sentences, *b* = .10, SE = .06, *t*(709.0) = 1.61, *p* = .108. However, there were no statistically significant fixed effect of Language, *b* = –.19, SE = .13, *t*(738.3) = –1.52, *p* = .129, or a Sentence type × Language interaction, *b* = –.10, SE = .12, *t*(709.0) = –.86, *p* = .389 (see **Tables 2** and **5,** and **Fig 7**).

#### 4.2.3. Stereotypicality ratings

The analysis of the stereotypicality ratings revealed a fixed effect of Sentence type, such that stereotypically incongruent sentences were rated as less stereotypical than stereotypically congruent, *b* = 3.62, SE = .04, *t*(705.3) = 95.57, *p <* .001, and semantically correct sentences, *b* = 2.07, SE = .04, *t*(719.2) = 54.17, *p <* .001. Moreover, stereotypically congruent sentences were rated as more stereotypical than semantically correct sentences, *b* = –1.55, SE = .04, *t*(717.3) = –40.46, *p <* .001. There was also a fixed effect of Language, such that English sentences were rated as more stereotypical than Polish sentences, *b* = .13, SE = .04, *t*(511.5) = 2.60, *p* = .009.

The analysis also revealed a Sentence type × Language interaction. Planned comparisons demonstrated that semantically correct, *b* = .16, SE = .06, *t*(856.6) = 2.46, *p* = .013, and stereotypically congruent sentences, *b* = .22, SE = .06, *t*(862.1) = 3.35, *p* < .001, were rated as more stereotypical in English than Polish. However, stereotypically congruent sentences were rated as similarly stereotypical in both languages, *b* < .01, SE = .06, *t*(862.0) = .04, *p* < .967 (see **Tables 2** and **5,** and **Fig 7**).

## 5. General discussion

The current contribution presents ratings for stereotypically congruent, stereotypically incongruent, semantically correct, and semantically incorrect sentences in Polish (Study I) and English (Study II). Besides providing the norms on how the four types of sentences are evaluated as far as their levels of meaningfulness, probability of use, and stereotypicality are concerned, the two studies show how respondents’ gender and gender schema (as reflected in Bem Sex-Role Inventory scores [23]) modulate their ratings.

Our findings from both studies confirmed that the sentences included in our database well represent the categories ascribed to them. First of all, in both studies, sentences that violate gender stereotypes (e.g., *Their nephew became a hairdresser immediately after graduating*.) were assessed as strongly violating common gender stereotypes, less probable to use in everyday language, and less meaningful relative to both sentences that adhere to gender stereotypes (stereotypically congruent sentences; e.g., *Their niece became a hairdresser immediately after graduating*.) and those classified as semantically correct (e.g., *There is only one hairdresser with such experience*.). This strongly shows that stereotypes are transmitted through language [9], and their violations exert influence on language perception.

Importantly, it needs to be noted that though stereotypically congruent and incongruent sentences differed only in whether they referred to a male or female, such a subtle manipulation of a sentence structure was impactful enough to modulate how participants rated sentence meaningfulness and the probability of use. While such an effect might not seem surprising as far as the probability of use is concerned, given that language violating gender stereotypes is typically of a lower frequency, the results observed within the meaningfulness scale are interesting, as they might potentially suggest that language users might perceive the violations of gender stereotypes as reflecting the violations of linguistic meaning, in line with previous research [10–12]. Taking into account that survey research provides insights into very overt language perception, it seems that language users consciously identify language information that is against stereotype-based expectations regarding males and females as *less meaningful* [14,15].

Another aim of the present research was to examine the potential role of individual characteristics (i.e., gender and gender schemas) in how raters evaluate the presented sentences. In both Study I and Study II, the analyses with gender schema types as a factor revealed that compared to other participants, sex-typed individuals provided consistently more stereotypical evaluations of the presented sentences. Such results are in line with the gender schema theory [23], according to which sex-typed individuals strongly adhere to their expected male or female gender roles and might consequently be more predisposed to use gender schemas to organize and process sex-typed information about themselves and others. This in turn might have led to their strong internalization of gender stereotypes, as a result of which they were more sensitive to stereotypes encoded in the present sentences.

On the other hand, less consistent results were observed as far as the role of participants’ gender is concerned. While in Study I, females rated stereotypically congruent sentences as more probable to be used in everyday language compared to males, in Study II, males rated stereotype incongruent sentences as more probable compared to females. Though previous research has pointed to a stronger internalization of gender stereotypes in females [1,24], this has only been revealed in Study I. More research is therefore needed to thoroughly investigate the role of participants’ gender in the perception of stereotype-laden language.

Finally, since the current research provides ratings for a set of sentences in both Polish and English, we also employed a between-language comparison to investigate potential differences influenced by the distinctive characteristics of each language system. This comparative analysis is particularly relevant due to the grammatical gender structure in Polish, where each noun is marked for gender, in contrast to English, which employs natural gender. We consequently assume that the distinction in language systems may lead to variations in the perception of gender stereotypes. Notably, natural gender languages, such as English, have been previously shown to enhance gender fairness by increasing the cognitive availability of mental representations of female exemplars compared to masculine generics [41]. However, contrary to our initial expectations, the between-language comparison revealed that, with the exception of stereotypically congruent conditions, all sentence types were rated as more meaningful and stereotype-laden in English than in Polish. This unexpected finding suggests that the nature of specific sentences in English, compared to Polish, may contribute to this divergence, especially since we made sure our database does not include any Polish-English translation equivalents. Further exploration is consequently needed to better understand the underlying factors driving these cross-language disparities in sentence evaluations.

In conclusion, the present database of gender-stereotyped sentences in both Polish and English holds immense relevance for researchers in the field. The studies have yielded a rich dataset of sentences matched across the dimensions of meaningfulness, probability of use, and stereotypicality. The importance of these normed sentences lies in their potential to significantly aid researchers in the design and execution of experiments related to gender stereotype processing. By offering a standardized set of stimuli that have been rigorously evaluated, this resource facilitates consistency across different laboratories and research projects. The adaptability of this database to other languages and cultures further enhances its utility, enabling cross-cultural comparisons of empirical findings on stereotype processing. In essence, the normed sentences provided in this article serve as a valuable tool for researchers, streamlining their work and promoting a more cohesive and comparable understanding of the dynamics surrounding gender stereotypes in language perception.

## 6. Conclusions

The present contribution shows that gender stereotypically incongruent sentences are consciously evaluated as less meaningful and probable to use relative to sentences that adhere to stereotype-driven expectations regarding males and females. This suggests that stereotype violations communicated through language exert influence on language perception. Furthermore, the results yielded a stronger internalization of gender stereotypes among sex-typed individuals, thus pointing to the crucial role of gender schemas in the sensitivity to gender stereotypical attributes. All in all, the present contribution aims to broaden researchers’ stimulus choices and allow for consistency across different laboratories and research projects on gender stereotype processing. The adaptation of this database to other languages or cultures could also enable a cross-cultural comparison of empirical findings on stereotype processing.
